# Central giant cell granuloma in the posterior region of mandible mimicking a fibro-osseous lesion and hemangioma: a case report

**DOI:** 10.1186/s13256-024-04571-7

**Published:** 2024-05-21

**Authors:** Salma Tabatabaei, Maryam Paknahad, Javad Garmabi, Farhad Ghorbani

**Affiliations:** 1 Department of Oral and Maxillofacial Radiology, School of Dentistry, Shiraz University of Medical Sciences, Shiraz, Iran; 2 Department of Oral and Maxillofacial Radiology, School of Dentistry, North Khorasan University of Medical Sciences, Bojnurd, Iran; 3 Department of Oral and Maxillofacial Surgery, School of Dentistry, Shiraz University of Medical Sciences, Ghasrodasht Street, Shiraz, 7144833586 Iran

**Keywords:** Cone beam computed tomography, Central giant cell granuloma, Hemangioma

## Abstract

**Background:**

A central giant cell granuloma (CGCG) is a benign, proliferative, intraosseous, and non-odontogenic lesion occurring primarily in children and young adults. On the histological level, it is characterized by numerous multinucleated giant cells scattered randomly throughout a sea of spindle-shaped mesenchymal stromal cells which are dispersed throughout the fibrovascular connective tissue stroma containing areas of haemorrhage. When it comes to radiographic features, CGCG can have an array of variations, ranging from well-defined expansile lesions to ill-defined and destructive lesions, with or without expansion.

**Case presentation:**

This case report reviews an 11-year-old Caucasian patient with a chief complaint of slow-growing swelling involving the right posterior mandibular region. The cone beam computed tomography (CBCT) revealed an ill-defined mixed lesion mimicking both fibro-osseous lesion and hemangioma. However, microscopic examination revealed multinucleated giant cells in a fibrous stroma suggestive of central giant cell granuloma.

**Conclusion:**

Our intent in reporting this case is to highlight the importance of thorough clinical, radiographical and histopathological examination for accurate diagnosis and therapeutic interventions as well as to emphasize the importance of taking different possibilities into consideration when examining bony swellings in the head and neck region.

## Introduction

The central giant cell granuloma (CGCG) is a non-malignant neoplasm that predominantly impacts the pediatric and young adult populations. CGCG is responsible for roughly 7% of all benign tumors in the jaws [1]. According to the World Health Organization, this particular intraosseous lesion is characterized by the presence of several areas of bleeding, clusters of multinucleated giant cells, and a certain degree of woven bone within the septae of fully developed fibrous tissue that traverses the lesion [2]. The incidence of CGCG has a greater frequency among the female population in comparison to males, while the age range of affected individuals typically spans from 10 to 25 years. The observed lesion typically exhibits midline crossing, with the body of the mandible anterior to the first molars being the most commonly affected region [3, 4]. CGCG has a diverse array of radiographic characteristics, which can range from clearly delineated expansile lesions to lesions that are poorly defined and exhibit destructive qualities, either with or without expansion [5].

This paper presents a case study detailing an atypical radiographic manifestation of central giant cell granuloma (CGCG) in the mandibular posterior region of an 11-year-old male patient.

## Case report

The oral and maxillofacial surgery department received a referral for a Caucasian patient who was 11 years old and presenting with swelling on the right side of the mandible. The patient's medical records revealed the onset of swelling approximately 18 months ago, which subsequently exhibited a progressive enlargement over time. Despite multiple previous visits, the lesion was overlooked due to low socio-economic status of the patient's family and their negligence. In spite of intermittent tenderness experienced at the location of the lesion, the patient did not report any pain. In addition to the aforementioned information, it should be noted that the patient exhibited a satisfactory overall state of health, devoid of any preexisting medical conditions or instances of physical injury. The patient's case lacked a medical record that provided any relevant information.

During the extraoral examination, a diffuse swelling was observed that extended from the right commissure of the mouth to the angle of the mandible in an anteroposterior direction. Additionally, the swelling extended from the right commissure of the mouth to the lower border of the jaw in a superoinferior direction. The overlying tissue covering the edematous area exhibited a typical appearance, devoid of any discernible secondary alterations or fistulous tracts. A pink-purplish area with an asymmetrical boundary was observed on the right side, spanning from the inferior margin of the nose to the superior margin of the upper lip. There were no limitations imposed on the act of mouth opening. No lymph nodes in the region were found to be palpable or painful.

**Fig. 1:**
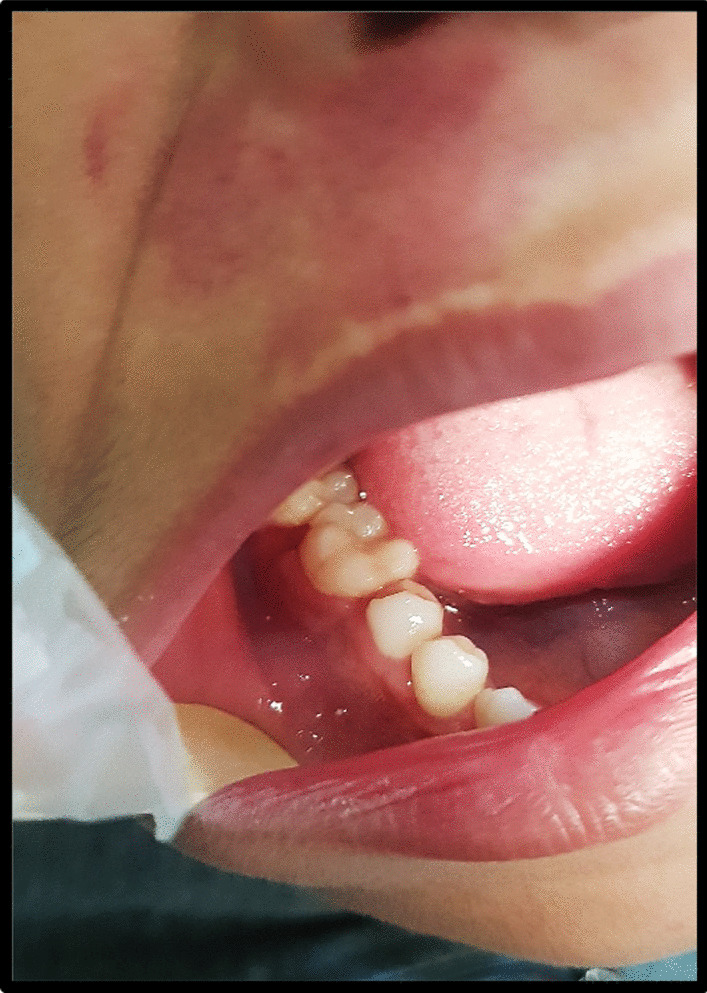
Intraoral picture showing swelling on the lower right vestibule and buccal mucosa

Intraorally, a swelling was observed, specifically on the right vestibule and buccal cortex of the jaw. The size of the swelling was estimated to be around 3–4 cm, and it extended from the right mandibular first premolar region to the distal aspect of the right second molar region. There was no observable evidence of significant enlargement on the lingual cortex. There was no observation of paresthesia. Upon palpation, the observed enlargement had a firm consistency like bone, with no tenderness or pulsation detected. The oral mucosa had a normal color and texture, without any presence of ulcers, secondary alterations, or sinus openings. The teeth located at the site of the lesion exhibited vital signs, were non-mobile, and showed no signs of decay (Fig. 1).

**Fig. 2:**
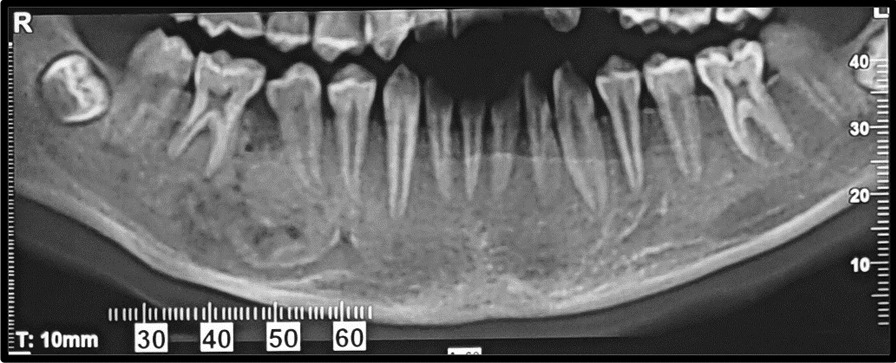
cone bean computed tomography image (panoramic reconstruction) revealed an ill-defined mixed lesion in the right side of the mandibular body extending from mesial aspect of right first premolar to periapical region of right first molar. Note the Inferior displacement of inferior alveolar canal and root divergence of 2nd premolar and 1st molar

**Fig. 3:**
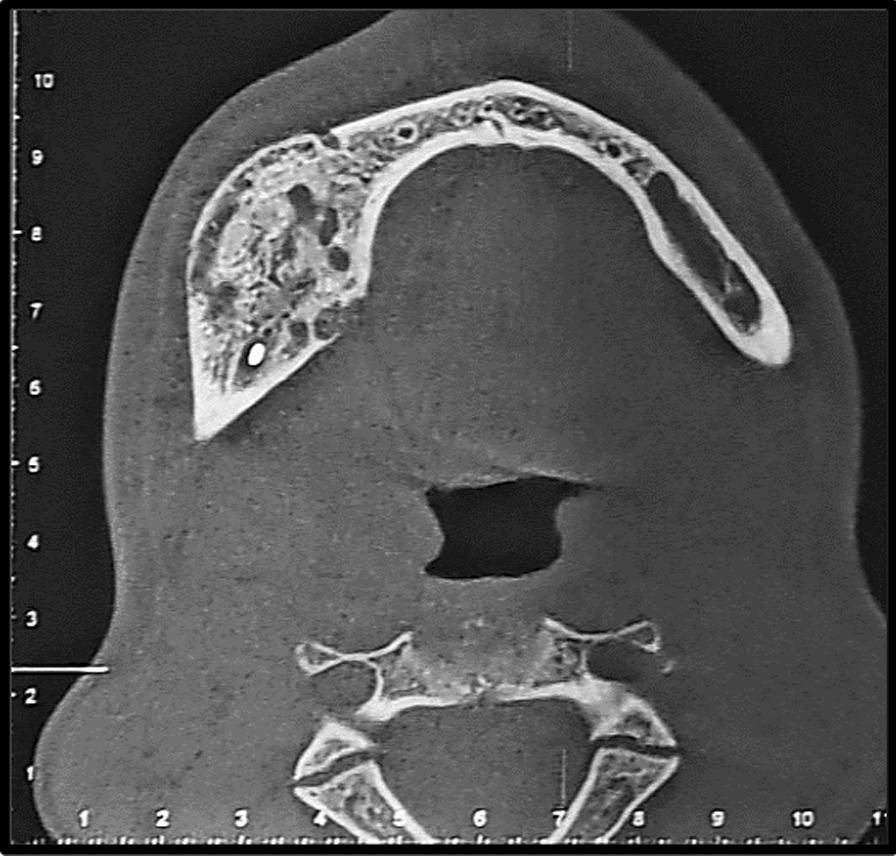
Cone bean computed tomography image (axial view) revealed expansion, thinning and loss of continuity of both buccal and lingual cortices

**Fig. 4:**
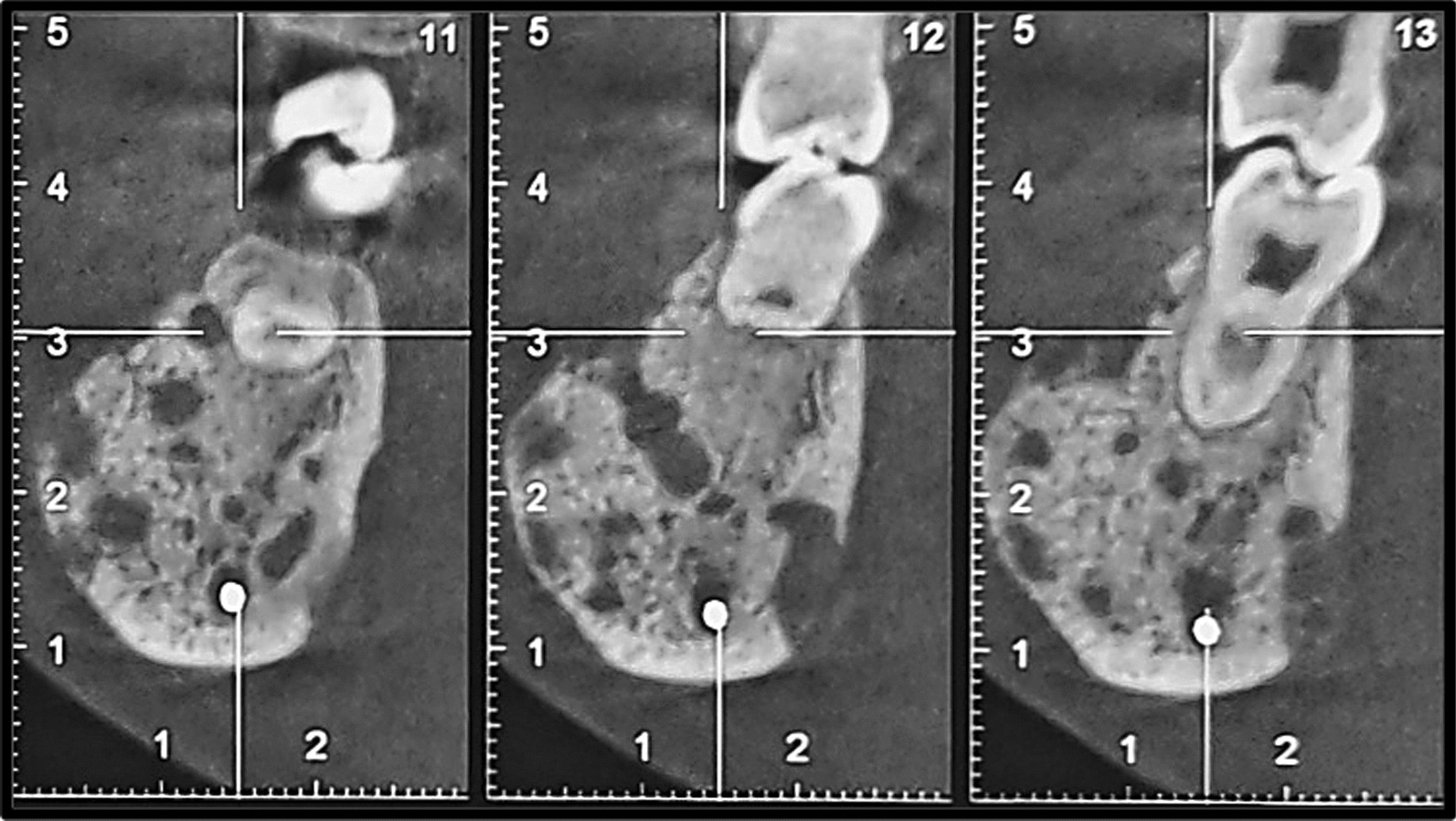
Cone bean computed tomography image (cross-sections). Erosion of alveolar crest, expansion, thinning and loss of continuity of both buccal and lingual cortices are evident

The CBCT examination revealed an expansile ill-defined mixed lesion located on the right side of the mandibular body, extending from the mesial aspect of the right first premolar to the periapical region of the right first molar. The presence of the lesion has resulted in the enlargement, thinning, and disruption of the buccal and lingual cortices. The presence of alveolar crest erosion, inferior displacement of the inferior alveolar canal (IAC), and root divergence of the second premolar and first molar could be observed. There was no evidence of root resorption observed in the teeth within the specified zone (Figs. 2, 3, 4). Differential diagnosis included juvenile ossifying fibroma, central hemangioma, and central odontogenic fibroma. Rule out of malignant transformation of juvenile ossifying fibroma was also recommended.

Upon observing the presence of a port wine stain on the right upper lip, along with radiographic characteristics such as the presence of lumen-like radiolucensies within a radiopaque background, the patient was referred for computed tomography angiography (CTA) assessment in order to exclude the presence of any vascular abnormalities prior to undergoing a biopsy procedure.

**Fig. 5:**
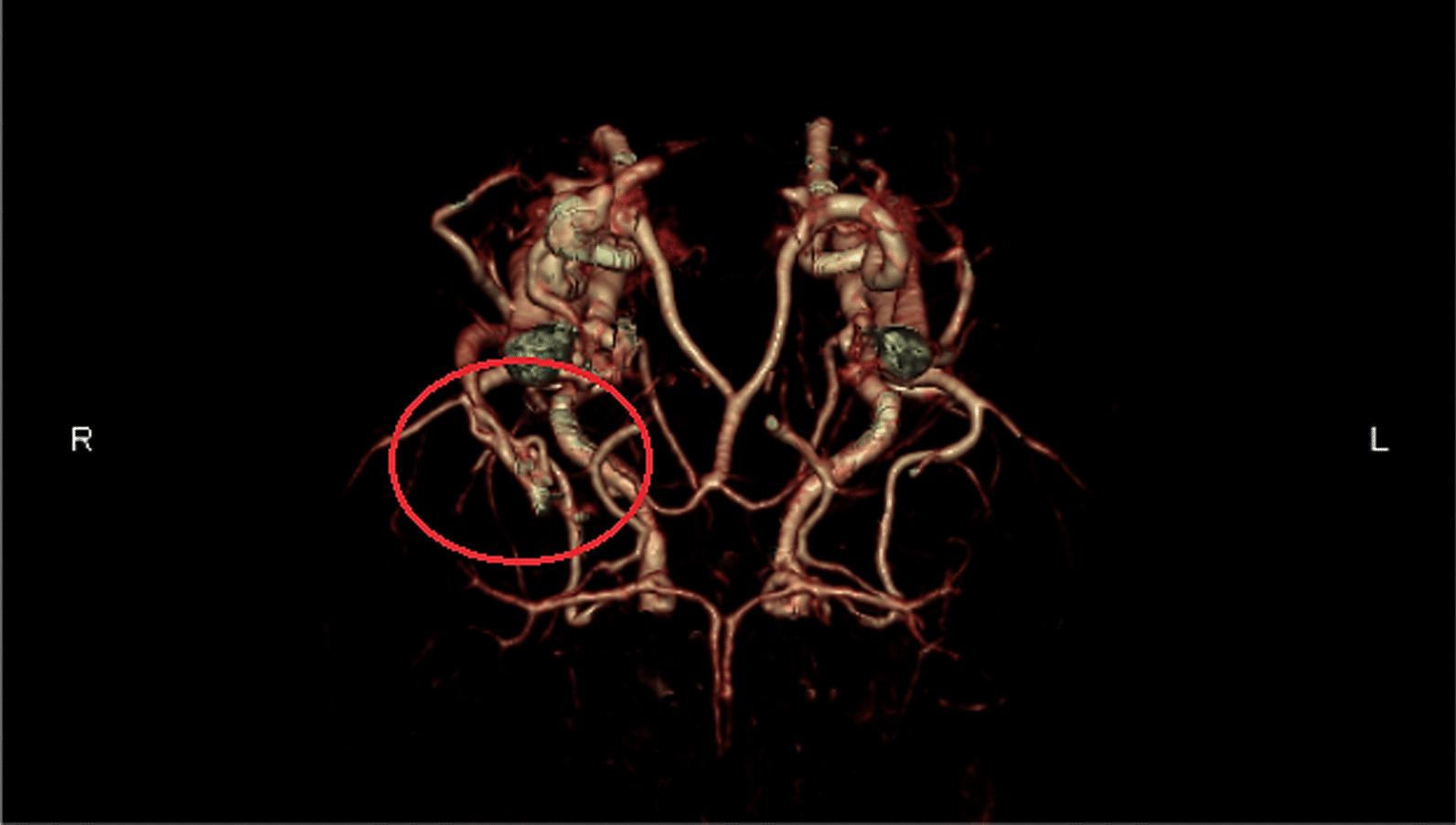
Contrast enhanced computed tomography angiography. Cranio-caudal 3D view showing vascular proliferation in the right side (red ring) comparing to the left side

A spiral computed tomography angiography (CTA) of the neck with intravenous contrast revealed the presence of an expansile mixed lytic lesion measuring approximately 20 × 35 mm in the right side of the mandible's body. Notably, there was no considerable vascularity observed in the lesion. The results of the catheter coronary angiography (CA), internal carotid artery (ICA), and external carotid artery (ECA) examinations indicated normal findings. The jugular veins exhibited normal characteristics bilaterally. Multiple lymph nodes were observed bilaterally in the cervical region, measuring up to 16 × 7 mm in the right posterior triangle. They were reactive in appearance (Fig. 5A-C). Based on the CTA impression, possibility of an intraosseous hemangioma (low flow) was considered. Other provisional diagnosis was fibrous dysplasia and osteoblastoma.

**Fig. 6:**
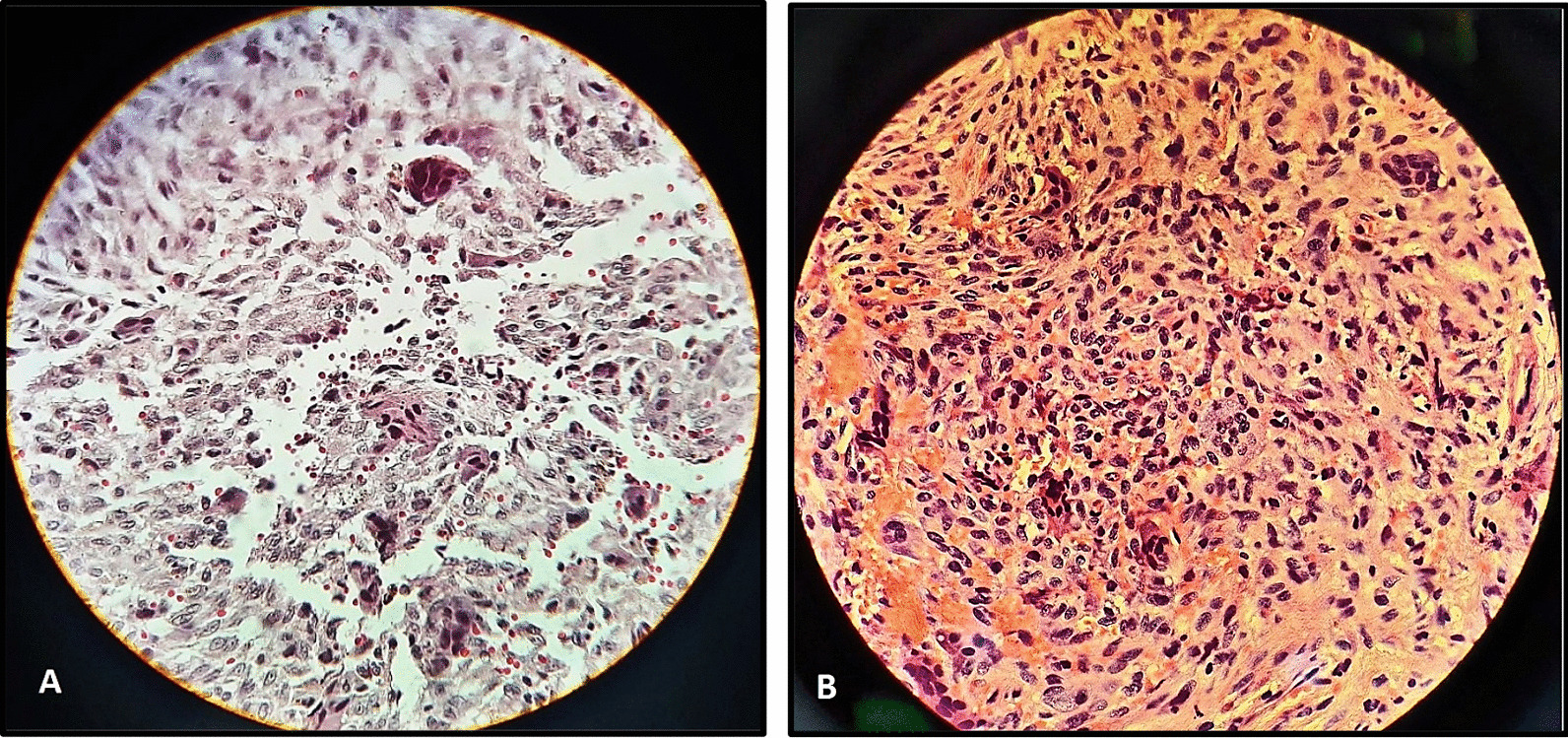
**A** connective tissue stroma with multinucleated giant cells. **B** blood-filled spaces separated by connective tissue septa containing fibroblasts, osteoclast type giant cells

An oral and maxillofacial surgeon with at least two decades of experience performed the incisional biopsy under local anesthesia. Upon surgery a dark brownish granulation tissue was found which was not hemorrhagic. Postoperatively, the patient recovered satisfactorily without any complications. The biopsy specimen was sent to department of oral and maxillofacial pathology. Gross examination revealed multiple pieces of irregular creamy brown elastic tissue measuring 1.5 × 1 × 0.2 cm in formalin solution. Under microscopic examination, giant cells were observed in an uneven cluster, with an intervening fibro-collagenous stroma and irregular trabecular bone ringed with osteoblasts. The histological findings were suggestive of central giant cell granuloma (Fig. 6A).

To exclude brown tumors of hyperparathyroidism, hematological testing was conducted on the patient to assess serum calcium, alkaline phosphatase, and parathyroid hormone levels. The findings were within normal limits. Hyperparathyroidism was thus excluded as a diagnosis.

Since pathological and radiographic findings contradicted each other, it was decided to repeat the biopsy. Following a period of three months, the patient underwent a second incisional biopsy under local anesthesia. There was no evidence of haemorrhage during the second biopsy as well. This time, the specimen was sent to a private pathological and clinical laboratory. On gross examination, two fragments of formalin immersed soft creamy-gray tissue measuring 1.6 × 104 × 0.8 cm was observed. On microscopic examination, it was found that there were a number of focal areas of hemorrhage and scattered clusters of multinucleated giant cells, bands of osteoid matrix within the connective tissue stroma, and anastomosing immature bone trabeculae (Fig. 6B). Based on the histologic features of the second biopsy, the diagnosis of central giant cell granuloma was confirmed. Three months clinical follow-up revealed satisfactory healing and no signs of relapse. No complications like wound dehiscence, infection, bone necrosis were seen on clinical examination.

## Discussion

Pediatric patients often experience facial swelling, which requires a thorough understanding of clinical and imaging presentations, as well as the common anatomical locations where pathologies occur [6]. In the field of dentistry, inflammation is a prevalent condition that often affects the jaws [7]. Acute swelling accompanied by inflammation and pain can be a sign of several medical conditions, including lymphadenitis, odontogenic infection, skin abscess, sinusitis, or osteomyelitis [1, 6]. In this particular case, the absence of any infectious sources, such as decayed teeth or signs of inflammation as determined through clinical and radiographic assessments, and afterwards confirmed through microscopic analysis of the biopsy sample, allowed ruling out inflammatory conditions as the underlying cause of the swelling.

In pediatric cases, the presence of gradual and limited expansion of facial swelling may indicate many conditions, such as neurofibroma, hemangioma, lymphangioma, vascular malformation, and fibro osseous lesions [6]. In the current scenario, the observed clinical presentation of a firm bony swelling with intact skin color and texture, along with the absence of any neurosensory impairments, did not support the likelihood of a diagnosis of neurofibroma, lymphangiomas, soft tissue hemangioma, or vascular malformation. However, it is important to note that there exist specific types of vascular malformations and Hemangiomas that have the potential to manifest within the bone (intraosseously). Furthermore, a limited number of investigations in the literature have shown the simultaneous presence of vascular malformations, such as port wine stains, and vascular malignancies [8, 9]. Considering the patient's port wine stain on the upper lip and the imaging characteristics of the lesion on cone beam computed tomography (CBCT), it was decided to examine the possibility of intraosseous hemangioma and vascular malformation prior to proceeding with a biopsy. This precaution was further necessary to avoid any irreversible sequelae. As a consequence, it was decided to recommend a computed tomography angiography (CTA) for the patient in which primary differential diagnosis was reported to be a low flow hemangioma.

Hemangiomas are vascular anomalies that are predominantly observed in pediatric populations. Generally, this phenomenon manifests itself during the second decade of life and exhibits a higher incidence rate among the female population compared to the male population. Additionally, there exists a central (intraosseous) variant, predominantly observed in the vertebrae and skull, with a relatively low incidence in the jaw [10]. A positive link exists between the occurrence of hemangioma in the mandible and the maxilla, with a ratio of two to one. The posterior body and ramus of the mandible exhibit a higher incidence of affliction compared to other regions. Most individuals diagnosed with intraosseous hemangioma typically do not manifest any noticeable symptoms. The process of bone enlargement is characterized by a gradual and slow progression, resulting in the development of a painless expansion of the jaw that takes place over an extended period of several months or even years. If the sensation of pain is experienced, it typically manifests as a throbbing sensation. Furthermore, there have been reports of gingival bleeding characterized by oozing or pulsatile bleeding, as well as observations of teeth becoming loosened or displaced, and the early exfoliation of primary teeth [7, 10, 11]. A hemangioma is commonly recognized as a "great mimicker" due to its tendency to resemble other intrabony lesions when observed during radiographic inspection [10]. Despite the benign nature of this lesion, failure to establish an accurate clinical and radiographic diagnosis may lead to potentially fatal bleeding and mortality [7, 10, 12].

It is relevant to highlight that in the current case, the patient was an 11-years-old exhibiting an asymptomatic slow-growing lesion in the posterior right region of the mandible. These characteristics align with the typical attributes of intraosseous hemangioma, hence reinforcing the differential diagnosis previously derived from computed tomography angiography (CTA). Despite the initial diagnosis by the CTA indicating a low flow hemangioma, subsequent microscopic inspection revealed the presence of distinct multinucleated giant cells with equally dispersed nuclei, which were consistent with central giant cell granuloma (CGCG). This finding consequently led to the exclusion of intraosseous hemangioma which was additionally corroborated by the lack of hemorrhage observed throughout the incisional biopsy procedure.

The fibro-osseous lesion of the oral and maxillofacial region, conversely, encompasses a diverse range of benign bone-related diseases characterized by the gradual replacement of bone tissue with fibrous connective tissue. This entity consists of ossifying fibroma, fibrous dysplasia, and cemento-osseous dysplasia. [1, 6, 13, 14]. The histological characterization of fibro-osseous lesions frequently involves the presence of irregular bony trabeculae exhibiting variable degrees of maturation, accompanied by the potential presence of osteoblastic rimming. Additionally, these lesions are characterized by a proliferation of fibroblastic spindle cells interspersed between the bony trabeculae [13, 14]. The current case exhibited notable radiographic resemblances to fibro-osseous lesions. However, the presence of multinucleated giant cells uniformly dispersed within the stroma, along with areas of hemorrhage and chronic inflammatory cells, eliminated the possibility of a definitive fibro-osseous diagnosis.

Previous studies have observed the presence of masses of reactive osteoclast-like giant cells in some metabolic diseases, including hyperparathyroidism. These masses histopathologically correspond to central giant cell granulomas (CGCG) [15]. Nevertheless, the patient's serum calcium and alkaline phosphatase levels within the normal range effectively excluded the possibility of hyperparathyroidism. In this regard, we reached the diagnosis of Central Giant Cell Granuloma (CGCG) by systematically eliminating alternative etiologies.

In 1953, Jaffe established the term "central giant cell granuloma" (CGCG) to describe a type of reparative giant cell granuloma [4]. Nevertheless, there exists a contentious discussion over the initial nature of CGC lesions, namely whether they are reactive or neoplastic in origin [7]. The aforementioned lesion accounts for fewer than 7% of the total number of benign tumors found in the jaw. Typically, it manifests as a painless mass that grows gradually and expands in size [16, 17]. One study reported that nearly 75% of patients diagnosed with GCGs are younger than thirty [18]. In a study that was conducted by Devi *et al*. it was found that GCGs are commonly occur in the second or third decades of life [19]. In the systematic review by Jordan Richardson et.al, out of 55 cases of GCG, the mean age of the patients was under 30 years [20]. These results are consistent with the findings in our study with an age of 11 years old boy. CGCG occurs with a small inclination towards females [7, 16]. Some studies have states that they are three times more likely to be afflicted than males [19, 21]. Jordan Richardson et.al reported an even sex distribution in a study of 55 cases of CGCG [20].

Certain researchers have classified CGCG into distinct categories based on their level of aggression. The severity of the lesions can be attributed to several aspects, including pain, paresthesia, root resorption, rapid development, cortical perforation, and a high recurrence rate after curettage [13]. CGCG exhibits a diverse range of imaging characteristics. It is well-documented that the vast majority of CGCGs manifests in the jaw [22–24]. One study has reported that CGCG are two to three times more common in the mandible compared to the maxilla [19]. These findings are consistent with the results of our study as the jaw and more specifically mandible, was the most common location for CGCG.

The circumference of CGCG exhibits a range of characteristics, including distinct growing lesions as well as indistinct damaging lesions. Lesions of the central giant cell (CGC) are characterized by a higher susceptibility to expanding bone and the displacement of anatomical structures [7]. Although not a consistent feature, central giant cell granuloma (CGCG) can be associated with significant root resorption and tooth displacement [1, 4, 5, 7, 17, 25, 26].

As previously mentioned, in terms of age and location, the lesion in this case was consistent with the findings of the previous studies. However, a notable challenge arose from the disparity observed between the radiographic internal structure of the present lesion and the common internal structure of central giant cell granuloma (CGCG) previously documented in the literature. The internal composition of CGCG exhibits significant variability, encompassing a spectrum of manifestations that range from small unilocular apical lesions to extensive and devastating multilocular lesions [5, 26]. Certain multilocular lesions display a subtle granular calcification pattern that may necessitate adjustments in imaging brightness for detection. In other cases, this granular bone pattern is accompanied by delicate wispy striations or septa [7]. In contrast, the current case exhibited a poorly defined mixed lesion with a prominent sclerotic background, giving it the appearance of a fibro-osseous lesion. Additionally, the presence of several lumen-like radiolucensies indicated a vascular lesion resembling a hemangioma. An additional complication in this particular case pertained to the absence of hemorrhage seen in the biopsy, which contradicted the diagnosis made using computed tomography angiography (CTA) and the radiographic features of the lesion. In 2022, Marti-Flich *et al*. also reported rare presentation of CGCG. A case in which a 25 year-old male presented with preauricular swelling and a premature occlusal contact on the molars. The lesion had radiological features of aggressiveness and a high metabolic uptake. Pathological examination of the biopsy showed focal hemosiderin deposition and small, unevenly distributed clusters of giant cells, which appeared to be in favor of an aneurysmal bone cyst. Due to aggressiveness of the lesion and its high metabolic uptake, the surgical resection was chosen as a treatment modality. However, the final pathological examination of the excised lesion concluded to a central giant cell granuloma to be the final diagnosis [27]. The presence of all these inconsistencies prompted us to do a further biopsy for the purpose of corroborating the ultimate diagnosis, since these three lesions; intraosseous hemangioma, fibro-osseous lesion, and CGCG require significantly different treatment approaches.

Intraosseous hemangioma often requires intralesional injection of sclerosing agents such as boiling water, sodium morrhuate, and sodium tetradecyl sulfate 72 h before resection which is usually followed by osseous reconstruction. Moreover, the surgery should be performed with extreme caution in a well-equipped medical centre since the lesion is hemorrhagic in nature and occasionally need external carotid artery ligation to prevent profound bleeding and further complications [28].

On the other hand, fibro-osseous lesions have different treatment modalities depending on their origins: being developmental, reactive, or dysplastic diseases or neoplasms. for instance Juvenile ossifying fibroma or cemento-ossifying fibroma are often treated with either enucleation or excisional methods while fibrous dysplasia cases are usually treated solely with recontouring and some are just routinely followed up after the diagnostic biopsy [29].

Most commonly used intervention for CGCG is curettage. It ranges from simple curettage to resection. En bloc surgical resection with a 5-mm margin has been the traditionally accepted modality reducing the chances of recurrence [4]. In addition intralesional steroid injection and calcitonin therapy, via subcutaneous injections and intranasal sprays, has been used successfully as conservative therapy (2).

During the second microscopic examination, the presence of evenly distributed giant cells was observed within the cellular stroma. This stroma consisted of nonreactive, spindle-shaped fibroblastic stromal cells. Additionally, regions of hemorrhage containing hemosiderin deposits, inflammatory cell aggregates, and areas of fibrosis and reactive bone formation were also identified. The histopathological observation, along with the absence of hemorrhage after the second biopsy, contributed to the conclusive diagnosis in support of CGCG.

CGCG lesions are commonly managed with surgical intervention, specifically by employing curettage or resection techniques. Nevertheless, considering the patient's age, a decision was made to abstain from conducting resection in order to avoid disfigurement of the face. Instead, a more conservative treatment approach was adopted, involving curettage and corticosteroid injections.

The overall prognosis of the patient was good and 3 months clinical follow-up revealed satisfactory healing and no signs of relapse. No complications like wound dehiscence, infection, bone necrosis were seen on clinical examination.

## Conclusion

In regards to radiographic features, central giant cell granulomas have a wide variety of radiographic appearances, ranging from radiolucent to mixed-density, which indicates the presence of internal granular bone deposits. In this case, however, CGCG had radiographic characteristics that mimicked fibro-osseous lesions and hemangioma. Having such unusual presentations underscores the importance of a thorough histopathalogical and radiographic examination. In addition, it encourages clinicians to consider a variety of possibilities when examining bony swellings in the neck and head regions in order to improve their management and prognosis of these conditions.

## Data Availability

All data generated or analyzed during this study are included in this published article.

## Abbreviations

CGCG: Central giant cell granuloma
CBCT: Cone beam computed tomography
CTA: Computed tomography angiography
